# The Prevalence of Dental Caries in Primary Dentition in 3- to 5-Year-Old Preschool Children in Northern China

**DOI:** 10.1155/2020/5315236

**Published:** 2020-05-10

**Authors:** Kaiqiang Zhang, Jian Li, Zhenfu Lu

**Affiliations:** ^1^Department of Preventive Dentistry, School and Hospital of Stomatology, China Medical University, Shenyang, China 110002; ^2^Liaoning Provincial Key Laboratory of Oral Diseases, Shenyang, China 110002

## Abstract

**Objectives:**

To investigate the prevalence and factors promoting caries in primary dentition of 3-5-year-old children in Northeast China.

**Materials and Methods:**

Data of 1,229 children aged 3 to 5 years were randomly selected. The caries prevalence and other indicators were assessed, and the results analyzed by SPSS. A questionnaire was also given to the children's guardians to ascertain the potential risk factors associated with caries.

**Results:**

The decayed, missing, and filled teeth (dmft) index in children aged 3, 4, and 5 years old was 3.17, 5.13, and 6.07, respectively, while the caries prevalence rate was 62.16%, 75.89%, and 87.28% accordingly. The incidence of caries among rural children was higher than that in urban areas. Regarding oral health awareness, it was found that parents in urban areas had more accurate perceptions of oral health problems. It was also noted that the children's brushing habits were worrying. Family economic and medical resources are not the main causes of serious dental caries in rural areas.

**Conclusions:**

The oral health status of children aged 3-5 years is not optimistic. Many parents have a low awareness of oral health. Strengthening the promotion of oral health knowledge is an effective way to change the situation.

## 1. Background

Along with the constant improvement of the economy, children's oral hygiene has been brought to the forefront by parents as well as scholars in the largest developing country, China [1, 2]. Since the 1980s, three large-scale surveys of oral hygiene epidemiology have been conducted in China, in 1983, 1995, and 2005, respectively. These data offer great help in understanding the current situation of Chinese children's oral hygiene and their behavioral habits, while the data also provide scientific evidence for the government to formulate relevant health policies [3]. According to researches, in addition to physical health, children's oral hygiene is of the utmost importance in children's mental health as well as in interpersonal communication [4, 5].

Liaoning Province, situated in the northernmost area of China, is the only province that is both coastal and on the border. It has a land area of 1480 thousand square meters, occupying 1.5% of the total land area of China. At the end of 2017, the number of permanent residents was 43.689 million, of which the 0-to-15-year-old proportion of the population was 4.843 million, accounting for 11.09% of the permanent residents. The third national oral hygiene epidemiology survey, in 2005 [6], demonstrated that the caries prevalence rate of 5-year-old children in urban and rural areas of Liaoning Province was 64.3% and 83.6%, respectively, while the rates of dmft were 3.29- and 5.35-fold higher than the national average.

With the implementation of “the Revitalization of the Northeast” strategy over the past decade, the economy of Liaoning Province has been boosted. However, there appears to be no literature on the status of children's oral hygiene nor on the parents' understanding of the issue. The objectives of this study were (1) to record the prevalence of dental caries, (2) to investigate the degree of parents' attention to children's oral health, and (3) to compare the differences between urban and rural areas and the reasons for the differences between them.

## 2. Methods

### 2.1. Respondents

The respondents were permanent residents between the ages of 3 and 5 years old in rural and urban areas (having lived for more than six months at the place in question). Informed consent forms were signed by the children's guardians and were kept for record. The children received oral examinations by qualified dentists while parents received questionnaires and were interviewed by the same dentists. Ethical approval was received from the Institutional Review Board at the Chinese Stomatological Association (201502002).

### 2.2. Sampling Method

Multistage stratified sampling was adopted in the study. In the first stage, two districts (city) and two counties (town) were randomly selected in Liaoning Province according to population proportion sampling [7]. The results included Donggang City (county-level city/town), Heping District of Shenyang City (city), Linghe District of Shenyang City (city), and Zhuanghe City (county-level city/town). During the second stage, three kindergartens were randomly selected in each county (district), and for the third stage, using cluster sampling, 3-, 4-, and 5-year-old children were randomly selected from every kindergarten. The sampling capacity was estimated in accordance with the formula *n* = deff(*u*_*α*_^2^*p*(1 − *p*)/*δ*^2^). The design efficiency deff = 2.5, the level of confidence *u*_*α*_ = 1.96, the margin of error *δ* = 10%, the expected caries prevalence rate was taken as 66%, based on the findings of the third national oral hygiene epidemiology survey in 2005 [6]. The number of samples for each kindergarten was calculated taking into account the stratification factors and nonresponse rate (estimated at 20%).

### 2.3. Training for Examination Personnel

All personnel had master's degrees or higher, as well as oral practitioner qualifications and more than three years' experience in oral clinic work. Before the on-site examination, both theoretical and clinical trainings were performed by the technical group responsible for the current fourth national oral hygiene epidemiology survey. Each examiner, together with a reference examiner, was requested to examine 10 to 15 respondents with the aim of assessing the consistency of their clinical examination. Consistency between examiners and reference examiners as well as consistency between examiners was monitored. The kappa value was from 0.8 to 0.89, which met the requirements of the examination. Three recorders were equipped with the necessary requirements. Questionnaire investigators were assessed after integrated training and were able to conduct questionnaires after qualification.

### 2.4. Standards and Contents of the Examination

An oral health survey was conducted with reference to the *Oral Health Surveys: Basic Methods* (Fifth Edition) [8] recommended by the World Health Organization (WHO). Using an appropriate light source, the CPI probe was used to check the children's caries in a comprehensive manner. The questionnaire consisted of 3 parts: (1) oral health-related behaviors: tooth-brushing behavior, sugar intake, history, and experience of dental examinations; (2) family factors: parents' dental knowledge and attitude; and (3) medical treatment behavior: reasons for delaying treatment.

Data entry and analysis were done using the Statistical Package for Social Sciences (SPSS ver. 19.0). For enumeration data, the Pearson chi-squared test was adopted. For continuous data, the following test methods were used. Binary logistic regression was performed to investigate the effects of the independent variables on the children's dental caries and behaviors. The significance level was set at 0.05.

## 3. Results

### 3.1. Statistics of Sampling Results

Using multistage stratified random sampling, a total of 1229 children between the ages of 3 and 5 years were enrolled in the study. These included 318 in Linghe District (urban), 324 in Heping District (urban), 284 in Zhuanghe City (rural), and 303 in Donggang City (rural). Among them, boys accounted for 51.1%, while girls accounted for 48.9%.

### 3.2. Caries Prevalence Rate and the Statistics of dmft

**Figure 1:**
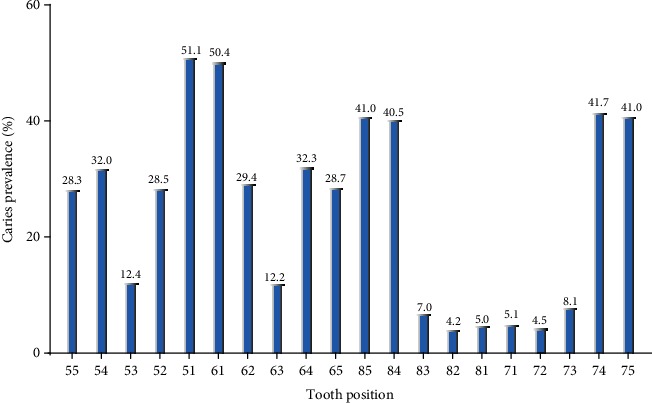
The caries prevalence of each tooth of children aged 3 to 5 years old.

**Figure 2:**
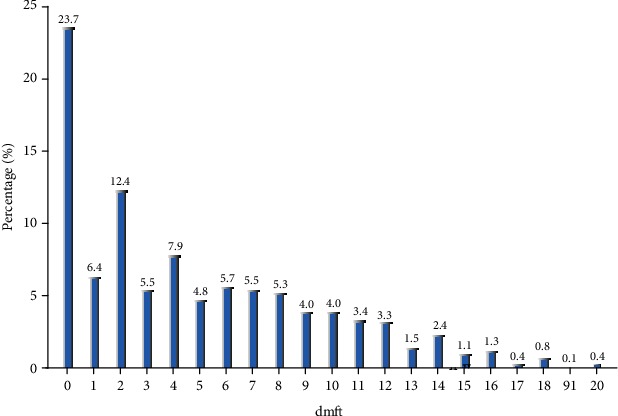
The frequency distribution of dmft.

Overall, the dmft index of children aged 3, 4, and 5 years old was 3.17 ± 4.073, 5.13 ± 4.793, and 6.07 ± 4.718, respectively, with corresponding caries prevalence rates of 62.16%, 75.89%, and 87.28%, respectively. As people grow up, the caries prevalence rate also increases. There were statistically significant differences in caries prevalence rates between the different age brackets (*P* < 0.05). The incidence of caries among rural children was significantly higher than that in urban areas (*P* < 0.05), while no significant differences were observed between boys and girls at different ages (*P* > 0.05) (Table 1). In the 5-year-old children, the prevalence of caries in urban and rural areas was 81.0% and 94.0%, respectively. In terms of caries filling, it was found that 2,074 children have 6,074 decayed teeth among them in total, including 5,351 unfilled caries, 25 missing caries, and only 398 filled caries, accounting for 6.55%.  Figure 1 shows the caries prevalence rate in different teeth of children aged 3 to 5 years old. The caries rate of the upper central incisor was the highest (51.08%) while the lower lateral incisor was the lowest (4.17%), and the rate of the lower mandible deciduous molar was higher than that of the upper mandible deciduous molar. In terms of the frequency of individual caries, 12.4% of children had two decayed teeth while children with four caries accounted for 7.9% (Figure 2). The caries were symmetrically distributed in the oral cavity in most cases.

### 3.3. Results of Questionnaires

The questionnaire was divided into attitude towards oral health, oral health awareness, children's eating habits, tooth-brushing behavior, and children's medical treatment. In terms of attitude, most parents believed that both oral hygiene and regular examination are of utmost importance. In addition, there was no significant difference between urban and rural areas; 98.6% of parents in urban areas and 98.8% of parents in rural areas believed that oral health is important, while parents who thought that the mother's bad teeth could affect children accounted for 40.5% and 29.0% of respondents in urban and rural areas, respectively. In terms of the 6-year-olds, no significant statistical difference was found between the answers of parents from urban and rural areas (Table 2). Regarding oral health awareness, eight questions were raised, including whether the bleeding gums are normal when brushing teeth, whether bacteria lead to caries, and whether the decayed deciduous teeth require treatment. On the whole, accurate perceptions tend to be associated with parents in urban, rather than rural, areas. We found statistically significant differences in accuracy in seven out of the eight questions. Regarding the protective effect of fluoride on teeth, accurate perceptions were higher in rural areas, although the difference was not statistically significant. In terms of eating before bedtime, 7.0% of urban children often ate desserts or had sweet drinks before going to bed, a higher percentage than that in rural areas (3.6%) and reflecting a statistically significant difference. In terms of brushing behavior, 81.3% of rural children had not started brushing their teeth before the age of two years and among children who had started brushing their teeth, only 15.0% brushed twice a day. Only 15.2% of parents assist their children aged 3-5 years old with tooth brushing. These statistics are far lower than those of urban children, resulting in statistically significant differences between urban and rural areas. Regarding issues such as using toothpaste when brushing, there were no significant differences, but fluoride toothpaste was only used in 22.9% and 28.2% of children in urban and rural areas, respectively. In rural areas, 45.7% of the children's parents were informed that their children had toothache. From the questionnaires, it was apparent that, of the children who had not seen the dentist in the previous 12 months, 58.4% of these parents did not consider that there might be a problem with their children's teeth. 16.7% of them knew that the children's gums were bad but not serious, while 13.3% thought that there was no need for their children to see dentists since the deciduous teeth will be replaced eventually. Economic reasons for not seeking medical treatment accounted for 0.2%, while reasons due to business accounted for 0.2% (Table 3). In particular, the results show that economic and medical resources are no longer the main factors affecting children's access to medical care in rural areas. Logical regression found that age differences, urban-rural differences, bedtime habits, and parents' educational differences were closely related to the incidence of dental caries in children (Table 4).

## 4. Discussion

Dental caries not only affect the function and appearance of teeth but may also become a repository for oral microorganisms [9] and may also cause systemic disease. Several mechanisms for this have been proposed, including the spread of the oral infection due to transient bacteremia resulting in bacterial colonization in extraoral sites, systemic infection caused by toxins secreted by oral pathogens, and systemic inflammation caused by soluble antigens from these pathogens [10]. Various diseases, including infective endocarditis [11], brain abscesses [12], and diabetes [13], have been linked to dental caries. These problems are caused by poor oral health; thus, monitoring the development of oral diseases is very important for children's health.

Using multistage stratified sampling, this study conducted an oral examination and parental questionnaires in an effort to understand the current situation of children's oral hygiene with 3-5-year-old children as the target population. A decade ago, a large-scale oral examination targeting 5-year-old children was developed, which revealed that the caries prevalence ratio in urban and rural areas was as high as 64.3% and 83.6%, respectively, while the dmft index was 3.29 and 5.35, respectively [6]. Ten years later, in association with the development of the Chinese economy, accompanied by improvements in living standards, it is apparent that great changes have taken place. The ratios have increased to 81.03% and 93.98% for urban and rural areas, respectively, while the mean dmft indexes have been raised to 4.73 and 7.51, respectively, which are higher than those of many developed countries and regions of developing countries, for instance, Pakistan, India, Europe, and the United States of America [14–19]. Compared with cities such as Shanghai and Guangzhou, it is worrisome that these values are still relatively high [20]. The main concerns are the increasing number of people suffering from caries; the increasing number of caries in children, as well as the early age; and the significant difference between urban and rural areas.

Compared with the survey in 2005 [6], this survey included the additional age group of children aged 3 and 4 years old. The findings showed that up to 75% of rural children suffered from caries at the age of three. It has been reported [21–23] that when caries occur at an early age, it will not only harm the children's oral health but also have a significant influence on their nourishment, mental health, and even quality of life. Early intervention during pregnancy and management of family behavior patterns are conducive to reducing the incidence of caries, particularly decreasing the incidence at preschool age, and thus enhancing their quality of life [24, 25]. Therefore, the prevention of dental caries should be instituted as early as possible; specifically, relevant measures ought to be taken in early pregnancy and after birth.

The position of caries was found to be, in a descending order of frequency, the maxillary anterior teeth, lower mandible deciduous molar, upper mandible deciduous molar, and mandibular anterior teeth. Two caries per child were found to be the most common, accounting for 12.4%. The deciduous molar has many deep fissures predisposing it to the formation of caries and subsequent loss. Monte-Santo et al. [26] observed that an early loss of molars produces a relatively large impact on oral health-related quality of life, which affects both children's physical and mental development. After a one-year follow-up, Honkala et al. [27] believed that pit and fissure closure is an effective means to prevent dental caries, especially for preschool children.

Fluoride toothpaste is an effective way to prevent caries and remove dental plaque, which has been widely recognized and actively promoted by researchers. According to surveys, in rural areas, 31.7% of rural children aged 3 and 5 years old still had not started brushing their teeth, while the number of children who brushed their teeth twice a day had increased by 7.5% to 15.0% compared to a decade ago [6]. About 15.2% of parents assist their children with tooth brushing. However, the usage rate of fluoride toothpaste was only 28.2%. These figures do seem to be better in urban areas. With the exception of the use of fluoride toothpaste, the other factors are higher than in the rural areas, showing statistically significant differences. Healthy habits contributed substantially to the relatively good performance in urban areas observed in the survey. Despite Oliveira et al.'s [28] views on the effect of fluoride in preventing caries in a short period of time, the majority of published studies [29, 30] indicate that a suitable amount of fluoride or fluoride toothpaste is able to prevent caries. Therefore, the popularization of fluoride toothpaste is an effective way to reduce the caries prevalence rate, especially in rural areas.

For the parental questionnaires, it was found that parents' oral health awareness has improved significantly in the past decade, especially in rural areas. For instance, the accuracy of the question “pit and fissure closure can prevent caries” increased from 7.6% to 39%, while the accuracy of “bleeding gums when brushing teeth is a normal phenomenon” increased from 53.3% to 71.4% [6], suggesting that rural residents now have a better understanding of oral health. However, compared with eating habits in urban areas, rural children tended to have more desserts and sweet drinks, which is also one of the reasons accounting for the caries prevalence in rural areas. In terms of medical treatment, the oral health-related questions posed to parents showed only two significant differences, namely, “whether suffered from toothache in the past 12 months” and “whether there is a dentist nearby.” However, only small differences were seen in responses to fees, medical conditions, time of treatment, and medical disinfection, suggesting that rural children are equipped with the basic medical essentials. Moreover, the relatively low outpatient rate is mainly due to parents' lack of oral health awareness. In terms of educational background, there are obvious differences between urban and rural parents, and this is closely related to the understanding of oral health.

**Figure 3:**
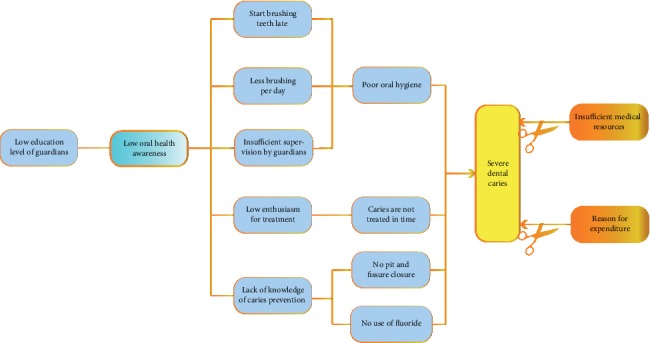
Factors affecting the development and treatment of dental caries in a Chinese population.

By comparing these results, it is not difficult to find that, compared with urban areas, children in rural areas start brushing their teeth at a later age, brush less often per day, have lower levels of parental involvement, and have insufficient oral disease prevention measures. These differences are the reasons why rural areas are higher in dental caries than urban areas. In particular, we would like to make it clear that, according to the results of our survey, the economic situation of the family and the distribution of medical resources are not the main reasons for the differences. The main reasons for these problems are the relatively low educational background of parents in rural areas and insufficient knowledge of oral health care (Figure 3). The results of this survey are different from people's previous perceptions of the medical situation in China. In the past, people thought that China was a developing country with a large population in rural areas and a lack of medical resources. However, in this situation, the problem is that, compared with their urban counterparts, rural residents do not pay sufficient attention to oral health, rather than being limited by economic conditions or a lack of medical resources.

In conclusion, the deciduous dental caries status of preschool children in North China was found to be still serious. Rural residents do not pay enough attention to oral health, a large number of children with oral problems fail to seek medical treatment in time, and parents do not fully realize the importance of oral health for children's growth. Strengthening oral health education for parents will be one of the most effective ways to improve the oral health of children aged 3 to 5, especially in rural areas.

## Figures and Tables

**Table 1:** Dental caries in children aged 3-5 years old.

Age	*N*	dmft			Caries frequency			dt	mt	ft
		Mean ± Sd	*t*-value	*P* value	Percentage	*χ* ^2^	*P* value			
3										
Urban	201	2.46 ± 3.712	-3.856	0.000	53.7	15.323	0.000	2.35 ± 3.632	0.00 ± 0.000	0.11 ± 0.537
Rural	132	4.24 ± 4.367			75.0			4.17 ± 4.315	0.01 ± 0.087	0.06 ± 0.344
Male	169	3.29 ± 4.316	0.553	0.580	60.4	0.476	0.490	3.17 ± 4.215	0.01 ± 0.077	0.11 ± 0.493
Female	164	3.04 ± 3.815			64.0			2.98 ± 3.800	0.00 ± 0.000	0.07 ± 0.445
Total	333	3.17 ± 4.073			62.2			3.08 ± 4.011	0.00 ± 0.055	0.09 ± 0.470
4										
Urban	209	3.68 ± 4.244	-6.284	0.000	64.1	29.705	0.000	3.23 ± 3.928	0.00 ± 0.069	0.45 ± 1.437
Rural	239	6.40 ± 4.894			86.2			6.23 ± 4.812	0.02 ± 0.129	0.15 ± 0.714
Male	230	5.56 ± 4.792	1.953	0.051	79.1	2.708	0.100	5.18 ± 4.675	0.02 ± 0.131	0.36 ± 1.290
Female	218	4.68 ± 4.763			72.5			4.45 ± 4.633	0.00 ± 0.680	0.22 ± 0.904
Total	448	5.13 ± 4.793			75.9			4.83 ± 4.664	0.01 ± 0.105	0.29 ± 1.120
5										
Urban	232	4.73 ± 4.206	-6.528	0.000	81.0	16.886	0.000	4.03 ± 3.962	0.02 ± 0.185	0.68 ± 1.652
Rural	216	7.51 ± 4.820			94.0			7.07 ± 4.680	0.07 ± 0.347	0.37 ± 1.105
Male	229	6.10 ± 4.782	0.133	0.894	85.6	1.201	0.273	5.54 ± 4.635	0.05 ± 0.306	0.51 ± 1.362
Female	219	6.04 ± 4.660			89.0			5.46 ± 4.527	0.03 ± 0.242	0.55 ± 1.484
Total	448	6.07 ± 4.718			87.3			5.50 ± 4.578	0.04 ± 0.277	0.53 ± 1.422

**Table 2:** The percentage of response on questions of oral health awareness and attitude.

	Urban			Rural			*χ* ^2^	*P* value
	Yes	No	Unknown	Yes	No	Unknown		
Awareness								
Gum bleeding is normal when brushing your teeth	10.6	84.0	5.5	18.9	71.4	9.7	28.217	0.000
Bacteria are one of the causes of inflammation of the gums	89.3	5.3	5.5	84.7	7.5	7.8	5.724	0.057
Cleaning teeth is not useful for preventing inflammation of the gums	11.5	74.8	13.7	22.1	58.4	19.4	39.142	0.000
Dental caries are caused by bacteria on teeth	85.5	4.8	9.7	76.1	5.6	18.2	20.069	0.000
Sweets can lead to tooth decay	88.2	7.0	4.8	81.8	9.0	9.2	11.509	0.003
Primary teeth do not require treatment	7.0	80.2	12.8	12.9	63.7	23.3	41.741	0.000
Pit and fissure sealant can prevent dental caries of children	48.6	5.1	46.3	39.0	10.4	50.6	18.650	0.000
Fluoride can prevent dental caries of children	8.9	40.8	50.3	12.4	42.9	44.6	6.075	0.048
Attitude								
Oral health is important to life	98.6	0.2	1.2	98.8	0.2	0.9	1.55	0.671
Regular oral examination is necessary	91.7	0.9	3.9	89.4	1.4	5.1	2.047	0.563
Teeth are born good or bad, no correlation with the protection	7.6	88.8	3.3	12.4	83.1	3.7	9.324	0.025
We should rely mainly on ourselves to prevent oral diseases	96.6	1.9	1.4	92.8	4.6	2.2	9.215	0.027
Protecting the first permanent molar is important	92.7	0.8	6.4	89.6	1.0	8.0	7.746	0.052
Mother's bad teeth can affect children	40.5	33.8	25.2	29.0	38.5	31.5	19.121	0.000

**Table 3:** Oral health behaviors of children surveyed and related factors affecting children's dental examination.

	Urban		Rural			*P* value
	*N*	%	*N*	%	*χ* ^2^	
Eating sweets before sleep						
Every day	45	7.0	21	3.6	18.577	0.000
Once in a while	350	54.5	386	65.8		
Never	247	38.5	180	30.7		
Brushing or not						
Yes	541	84.3	401	68.3	43.607	0.000
No	101	15.7	186	31.7		
The age of starting tooth brushing						
Before 1 year of age	61	11.3	24	6.0	116.594	0.000
Before age of 2 years	196	36.2	51	12.7		
Before age of 3 years	214	39.6	175	43.6		
Before age of 4 years	70	12.9	221	23.5		
Brushing (times per day)						
Twice daily or more	171	31.6	60	15.0	48.546	0.000
Once daily or more	314	58.0	251	62.6		
Helping children brush teeth						
Not brushing teeth every week	56	10.4	90	22.4		
Every day	176	32.5	61	15.2	56.460	0.000
Several times per week	19	3.5	9	2.2		
Once in a while	298	55.1	542	57.5		
No	48	8.9	135	14.3		
Brushing teeth with toothpaste						
Yes	520	96.1	387	96.5	1.304	0.521
No	13	2.4	6	1.5		
Unknown	8	1.5	8	2.0		
Use of fluoride toothpaste						
Yes	119	22.9	109	28.2	40.283	0.000
No	207	39.8	78	20.2		
Unknown	194	37.3	200	51.7		
Discomfort in the past year						
Never	389	60.6	294	50.1	13.785	0.003
Sometimes	207	32.2	241	41.1		
Often	25	3.9	27	4.6		
Unknown	21	3.3	25	4.3		
Used to check teeth	222	34.6	186	31.7	1.157	0.282
Did not check teeth due to						
Teeth are not causing problems	265	57.1	362	58.4	0.166	0.683
Teeth are not very bad	72	15.5	75	16.7	0.224	0.636
No need to check primary teeth	76	16.4	60	13.3	1.673	0.196
High cost	1	0.2	1	0.2	0.000	0.983
Inconvenient	6	1.3	5	1.1	0.064	0.801
Busy work	18	3.9	17	3.8	0.006	0.936
Child afraid of dentist	31	6.7	31	6.9	0.016	0.901
No dentist nearby	2	0.4	10	2.2	5.657	0.017
Fear of infectious diseases	10	2.2	8	1.8	0.169	0.681
Distrust dentist	19	4.1	14	3.1	0.635	0.425
Kindergarten provides dental examination	39	8.4	40	8.9	0.068	0.795
Parents' education						
Junior high school and below	112	17.4	391	66.6	395.032	0.000
Senior middle school	119	18.5	126	21.5		
University and above	411	64	70	11.9		

**Table 4:** Binary logistic regression analysis for the dental caries status.

	*B*	S.E.	Wals	df	Sig.	Exp (*B*)	95% C.I.
Age	0.774	0.107	52.750	1	0.000	2.169	1.76-2.673
Urb_rur	-0.834	0.205	16.503	1	0.000	0.434	0.29-0.649
Eating sweets before sleep	-0.282	0.144	3.846	1	0.050	0.754	0.569-1
Parents' education	-0.110	0.054	4.130	1	0.042	0.896	0.806-0.996
Constant	-0.652	0.635	1.055	1	0.304	0.521	

## Data Availability

The calculated data by statistical software (spss 19.0) used to support the findings of this study are included within the article. The original data includes the name, date of birth, ID card code, and other illnesses of respondents, etc., in order to respect the privacy of the respondent. Data are available from [Zhenfu Lu, zflu2006@163.com] for researchers who meet the criteria for access to confidential data.
